# What about It?

**Published:** 1890-04

**Authors:** 


### What About It?

From present indications it looks as though the great World's Exposition, to be held in Chicago, in 1892, will not occur till 1893, or some other year. Then of course, the International

Dental Congress will stand over also. It is well to have time enough for important matters.

